# Analysis of secondary growth in the Arabidopsis shoot reveals a positive role of jasmonate signalling in cambium formation

**DOI:** 10.1111/j.1365-313X.2010.04283.x

**Published:** 2010-07-14

**Authors:** Eva M Sehr, Javier Agusti, Reinhard Lehner, Edward E Farmer, Martina Schwarz, Thomas Greb

**Affiliations:** 1 Gregor Mendel Institute of Molecular Plant Biology, Austrian Academy of SciencesDr Bohr-Gasse 3, 1030 Vienna, Austria; 2 Department of Plant Molecular Biology, University of LausanneBiophore, CH-1015 Lausanne, Switzerland

**Keywords:** secondary growth, cambium, *JAZ10/TIFY9/JAS1*, jasmonate, mechanostimulation, lateral meristem

## Abstract

After primary growth, most dicotyledonous plants undergo secondary growth. Secondary growth involves an increase in the diameter of shoots and roots through formation of secondary vascular tissue. A hallmark of secondary growth initiation in shoots of dicotyledonous plants is the initiation of meristematic activity between primary vascular bundles, i.e. in the interfascicular regions. This results in establishment of a cylindrical meristem, namely the vascular cambium. Surprisingly, despite its major implications for plant growth and the accumulation of biomass, the molecular regulation of secondary growth is only poorly understood. Here, we combine histological, molecular and genetic approaches to characterize interfascicular cambium initiation in the *Arabidopsis thaliana* inflorescence shoot. Using genome-wide transcriptional profiling, we show that stress-related and touch-inducible genes are up-regulated in stem regions where secondary growth takes place. Furthermore, we show that the products of *COI1*, *MYC2*, *JAZ7* and the touch-inducible gene *JAZ10*, which are components of the JA signalling pathway, are cambium regulators. The positive effect of JA application on cambium activity confirmed a stimulatory role of JA in secondary growth, and suggests that JA signalling triggers cell divisions in this particular context.

## Introduction

Secondary or lateral growth is mediated by the activity of the vascular cambium. The cambium is an internal meristematic tissue that functions as a stem cell niche and is organized in a tube-like domain encompassing the growth axes. Compared with our understanding of the molecular control of apical meristem function and despite its essential role in many aspects of plant growth, the accumulation of biomass and wood formation, knowledge on molecular regulation of the vascular cambium is limited. This lack of knowledge is partly due to the fact that the tissue is not accessible by genetic approaches in most species, and that its dynamics have not been well characterized in models such as Arabidopsis. Although there is ample evidence for secondary growth in the Arabidopsis shoot, hypocotyl and root (Dolan *et al.*, 1993; Lev-Yadun, 1994; Busse and Evert, 1999; Altamura *et al.*, 2001; Chaffey *et al.*, 2002; Little *et al.*, 2002; Ye *et al.*, 2002; Ko *et al.*, 2004; Melzer *et al.*, 2008; Sibout *et al.*, 2008), the degree and dynamics of secondary growth have yet to be explored in detail. Such research is fundamental if Arabidopsis is to be established as a model for analysing the process of secondary growth at the molecular level and if participating signalling pathways are to be characterized.

**Figure 1:**
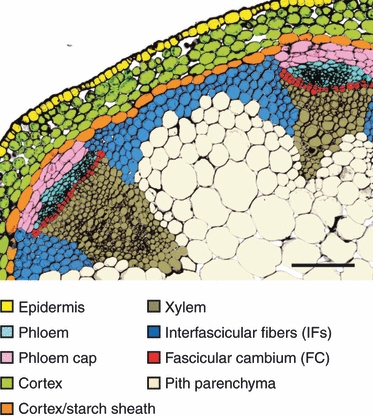
Tissue composition within the primary stem.No IC has been established in the interfascicular region. However, the FC in vascular bundles has started to produce secondary vascular tissue, which is visible as radial cell files in the xylem area proximal to the cambium (most prominent in the left-hand bundle). All other tissues are of primary origin. Scale bar = 100 μm.

In dicotyledonous plants, including Arabidopsis, initiation of cambial activity in shoots starts in a predetermined region of the vascular bundles, the fascicular cambium (FC, Figure 1). From there, it extends to interfascicular regions, where differentiated cells regain the ability to divide. In this way, the interfascicular cambium (IC) is established. This connects the FC of adjacent vascular bundles, creating a tube-like domain of meristematic activity. Histological analyses suggest that, in Arabidopsis, the IC is only established at the base of the stem and at nodal regions, and that most elongated internodes lack such a tube-like domain of meristematic activity (Little *et al.*, 2002). Various origins of the IC in various parts of the elongated stem have been reported. In nodes, interfascicular parenchyma cells appear to serve as cambium precursors, whereas the starch sheath, which is the innermost layer of the cortex (Figure 1), serves as the origin of the IC at the stem base (Altamura *et al.*, 2001; Ye *et al.*, 2002). The genes *COV1* and *HCA2* were isolated based on their role in IC regulation (Parker *et al.*, 2003; Guo *et al.*, 2009). *COV1* encodes a putative membrane protein with unknown function, and *HCA2* encodes a Dof transcription factor. *COV1* and *HCA2* are negative and positive regulators of IC formation, respectively; however, their up- and downstream factors are still unknown (Parker *et al.*, 2003; Guo *et al.*, 2009). A further mutant, *hca*, which is not allelic to *HCA2* and *COV1*, displays enhanced IC formation, but its molecular identity is not known (Pineau *et al.*, 2005).

Whether the initiation of cell divisions next to the FC depends solely on signalling molecules or also on the generation of tissue tension has been a matter of debate (Steeve and Sussex, 1989). On the one hand, a large number of papers have discussed hormonal control of the shoot cambium in various species (reviewed by Elo *et al.*, 2009), and polar transport of auxin, in particular, has been identified as an essential stimulus for (pro)cambium initiation and activity (Snow, 1935; Little *et al.*, 2002; Scarpella *et al.*, 2006; Wenzel *et al.*, 2007; Nilsson *et al.*, 2008; Donner *et al.*, 2009). In addition, cytokinins play an essential role in cambium formation and activity (Matsumoto-Kitano *et al.*, 2008; Nieminen *et al.*, 2008; Hejatko *et al.*, 2009), and gibberellins and ethylene have modulating activities (Björklund *et al.*, 2007; Love *et al.*, 2009). On the other hand, mechanical stimuli and constraints have a tremendous impact on developmental processes, one of which is secondary growth (Ko *et al.*, 2004; Hamant *et al.*, 2008; Chehab *et al.*, 2009).

Here, we characterize the dynamics of cambium initiation and activity in interfascicular regions in the elongating Arabidopsis shoot as an essential step during establishment of secondary growth in plants. Based on histological analyses and genome-wide transcriptional profiling, we hypothesize that intra-tissue tensions are involved in the regulation of these processes. By identifying the products of the genes *JAZ10*, *JAZ7*, *MYC2* and *COI1* and jasmonic acid (JA) itself as regulators of IC initiation and activity, we have identified a connection between JA signalling and secondary growth regulation. Based on these findings, we discuss a putative signalling cascade connecting mechanostimulation and meristem activation.

## Results

### IC formation in Arabidopsis progresses acropetally

Systematic analysis of the dynamics of secondary growth in the Arabidopsis inflorescence shoot is a prerequisite for identifying and characterizing participating signalling pathways. We undertook such an analysis by exploring the establishment of cambium activity in interfascicular regions because this is a prominent and easy to follow marker for secondary growth initiation. By performing histological analyses, we observed that, along the main inflorescence stem apically from the rosette, IC activity is initiated exclusively at the stem base and at the base of side shoots emerging from the axils of cauline leaves. In the latter case, IC formation barely extends into the main shoot (Figure S1). We therefore concentrated our investigations on the base of the main inflorescence stem, immediately above the uppermost rosette leaf, which, for simplicity, is denoted as the stem base throughout this paper. Stems 2, 5, 15 and 30 cm in height were subjected to histological analysis, and the cellular patterning in interfascicular regions was examined.

**Figure 2:**
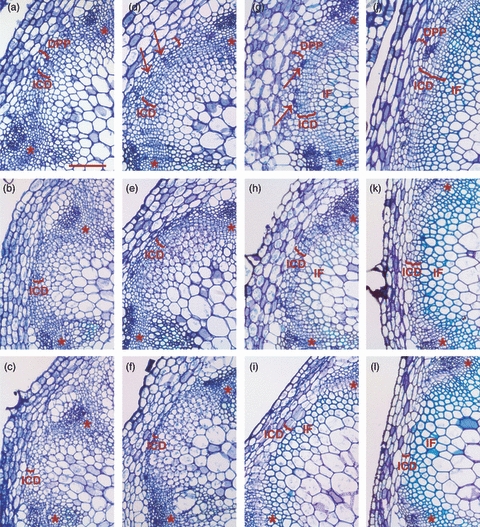
Histological analysis of the basal stem segment at various developmental stages.(a–c) 2 cm stems sectioned immediately above the rosette (a), and 1 mm (b) and 2 mm (c) above the rosette.(d–f) 5 cm plants analysed immediately above the rosette (d), and at 1.8 mm (e) and 3.7 mm (f) above the rosette. Phloem initiation is indicated by arrows (d).(g–i) Sections of 15 cm stems from immediately above the rosette (g), and from 2 mm (h) and 5.2 mm (i) above the rosette. Phloem initiation is indicated by arrows (g).(j–l) Sections of 30 cm stems taken from immediately above the rosette (j), and from 2.7 mm (k) and 7.1 mm (l) above the rosette.IFs, interfascicular fibres; ICD, interfascicular cambium-derived tissue (including IC); DPP, distal pith parenchyma. Asterisks indicate primary vascular bundles. Scale bar = 100 μm; same magnification throughout.

**Figure 3:**
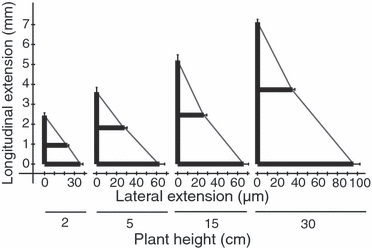
Quantitative analysis of the longitudinal and lateral extension of the IC and the ICD tissue at various developmental stages.

A defined and continuous zone, displaying periclinal cell divisions and connecting the FC of adjacent vascular bundles, was present in interfascicular regions at the very base of 2 cm stems (Figure 2a). Based on these characteristic cell divisions, the formation of radial cell files and the production of secondary vascular tissue (see below), we classified the cell division zone as the IC, and the IC together with tissues derived from it as IC-derived tissue (ICD). At the stage when the stems were 2 cm tall, the ICD consisted of 3 or 4 cells in radial orientation, located 3–5 cells proximal to the cortex (Figures 2a and S2). Cells between the ICD and the cortex were classified as pith parenchyma cells based on their shape and because they were not organized in radial files (Figures 2a and S2). At this stage, periclinal cell divisions in interfascicular regions were identified up to approximately 2.4 mm above the uppermost rosette leaf (Figure 3). In comparison to the very base of the stem, the ICD was closer to the cortex in more apical positions and was directly juxtaposed to it from a position of approximately 0.6 mm above the rosette (Figures 2b,c and S2). At the very base of 5 cm stems, the ICD had extended laterally to 7 or 8 cells (Figure 2d). At this stage, we observed clusters of cells, originating from cell divisions without a common orientation, distally to the IC. These were classified as differentiating phloem tissue, as they expressed the phloem-specific *APL:GUS* marker (Bonke *et al.*, 2003) (Figures 2d and S3a,b, arrows). IC initiation had progressed acropetally to 3.5 mm above the rosette, and, similarly to the 2 cm stems, was observed closer to the cortex in more apical parts of the IC-initiating segment (Figures 2e,f, 3 and S2). In 15 cm stems, IC initiation extended up to 5.1 mm above the rosette, and islands of phloem tissue could be clearly identified at the stem base (Figure 2g, arrows). At this stage, the presence of prominent secondary cell walls proximal to the ICD along the whole stem segment indicated the differentiation of pith parenchyma cells into interfascicular fibres (IFs) (Figures 2g–i and S2). Another change in comparison to 5 cm stems was that cell divisions in interfascicular regions were not only observed in pith cells but also in the starch sheath at the upper margin of the IC-initiating stem segment (Figures 2i and S2). In 30 cm stems, the extension of the stem segment with interfascicular cell divisions had progressed acropetally to 7.1 mm (Figures 2j,k and 3). At this stage, cell divisions in the starch sheath were found in a region from approximately 4.5–7.1 mm (Figure 2l).

Taken together, our analysis shows that there is an acropetal progression of IC initiation, and that secondary vascular tissue is produced in interfascicular regions of the Arabidopsis shoot. However, in contrast to shoot elongation, which progresses in an almost linear fashion from 2 to 30 cm (Figure S3c), the acropetal progression of IC formation decelerates (Figures 3 and 5a), showing that there is no linear correlation between shoot elongation and IC formation in Arabidopsis. During acropetal progression, the position of IC initiation is gradually shifted towards the cortex, and, in later growth stages, takes place in the cortex itself in the upper region of the IC-initiating stem segment.

### Transcriptional profiling links genes involved in mechanical stress signalling with secondary growth

We sought to elucidate the signalling pathways involved in IC initiation by identifying genes that were differentially expressed comparing primary and secondary stem segments. Analyses of gene expression profiles in various parts of the elongated Arabidopsis shoot and at various developmental stages have been performed previously (Oh *et al.*, 2003; Ko and Han, 2004; Ko *et al.*, 2004; Ehlting *et al.*, 2005). However, a specific comparison of stem segments with and without secondary growth, defined on the basis of a detailed histological analysis, has not yet been reported. We thus performed genome-wide transcriptional profiling comparing the lowermost 0.5 cm of the stem with a 0.5 cm segment 1.5 cm above the uppermost rosette leaf. Both samples were taken from the first internode of 15 cm plants (Figure S3d). In three biological replicates, 74 genes were identified as preferentially expressed in the ‘internode’ sample and 92 genes as preferentially expressed in the ‘base’ sample (fold change ≥ 1.8, *P*≤0.1; Tables S1 and S2). To confirm the reliability of these results, we checked the expression of 15 of the identified genes using RT-PCR, and were able to confirm the relative expression levels in all cases (Figure S4).

A significant proportion of the 92 genes preferentially expressed in the stem segment undergoing secondary growth overlap with gene sets identified in previous studies as either being up-regulated in mature versus immature stems (Ko and Han, 2004) or after induction of secondary growth by repeated removal of inflorescences (Oh *et al.*, 2003) or by weight adherence (Ko *et al.*, 2004, Table S2). This shows that our analysis is robust, and suggests that expression of a large number of previously identified genes differs not only temporally but also spatially along the stem.

**Figure 4:**
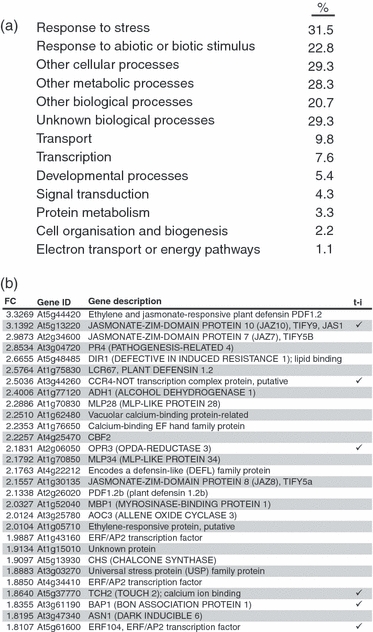
Stress-related genes are over-represented in the group of genes preferentially expressed at the stem base.(a) Biological function of genes identified as preferentially expressed at the stem base. Percentages add up to more than 100 because genes may belong to more than one functional category.(b) Stress-related genes preferentially expressed at the stem base. Ticks indicate whether genes were classified as touch-inducible according to Lee *et al.* (2005).

Previous studies have suggested an influence of mechanical stress on secondary growth-related gene expression in Arabidopsis shoots (Ko *et al.*, 2004). It is therefore tempting to speculate that up-regulation of genes at the stem base partially reflects the mechanical stress that cells experience due to tissue expansion, increasing shoot weight or wind-induced shoot movement (Hejnowicz, 1980; Ko *et al.*, 2004). This is supported by the identification of genes such as *TOUCH2*, which is inducible by mechanical stimuli (Braam and Davis, 1990; Ko *et al.*, 2004), or *EXPANSIN L1*, which is associated with relaxation of mechanical stresses in cell walls (Table S2) (Sampedro and Cosgrove, 2005). Interestingly, 32% of genes preferentially expressed at the stem base are classified as being stress-related according to gene ontology analyses (http://www.arabidopsis.org/tools/bulk/go/index.jsp) (Figure 4a). To assess the possibility that mechanical stimuli influence gene expression at the base of the stem, we compared our group of genes identified as preferentially expressed in the base of the stem with the group of touch-inducible genes identified previously (Lee *et al.*, 2005), and found that 20% of the genes identified as preferentially expressed at the stem base are also classified as being touch-inducible (Table S2), but none of them belong to the group of touch-repressible genes.

### JA signalling positively influences secondary growth

Some of the genes with the greatest difference in expression between the two stem samples analysed encode JA signalling components, and more than 8% of the identified genes are related to JA signalling or response (Figure 4b and Table S2). In view of this over-representation, which suggested a role for JA signalling components in secondary growth regulation, we analysed *JAZ10/JAS1/TIFY9* (At5g13220) and *JAZ7*/*TIFY5B* (At2g34600), the two most differentially expressed JA signalling components in our list of genes preferentially expressed in the stem base (Figure 4b and Table S2).

Elucidation of the molecular mechanism of action of *JASMONATE ZIM-DOMAIN* (*JAZ*) genes as repressors of JA signalling is a recent breakthrough in deciphering the JA signalling pathway (Chini *et al.*, 2007; Thines *et al.*, 2007; Yan *et al.*, 2007). JAZ proteins bind directly to the key transcription factor MYC2, and thereby prevent JA-dependent gene transcription (Chini *et al.*, 2007; Pauwels *et al.*, 2010). After jasmonate-isoleucine (JA-Ile) binds to COI1, the JA-Ile receptor that functions as the F-box component of the SCF^COI1^ complex, JAZ proteins undergo proteolytic degradation, allowing expression of MYC2-regulated genes (Chini *et al.*, 2007; Thines *et al.*, 2007; Chung and Howe, 2009; Yan *et al.*, 2009). In addition, eight of the 12 JAZ genes, including *JAZ10* and *JAZ7*, have been shown to be specifically JA-inducible (Chini *et al.*, 2007; Thines *et al.*, 2007; Yan *et al.*, 2007). The repression of JA signalling by JAZ proteins on the one hand and the JA inducibility of JAZ gene transcription on the other hand ensures the establishment of fine-tuned JA signalling in the presence of particular stimuli (Chung *et al.*, 2009). In particular, *JAZ10* has previously been implicated in growth responses related to JA signalling (Yan *et al.*, 2007).

**Figure 5:**
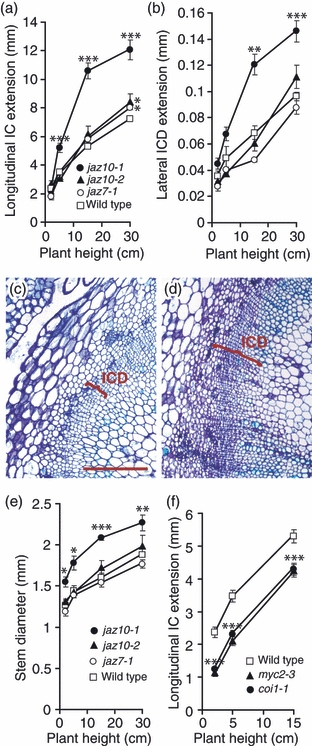
Secondary growth phenotype of JA signalling mutants.(a,b) Comparison of acropetal progression of IC initiation (a) and lateral ICD extension at the stem base (b) between wild-type, *jaz10-1*, *jaz10-2* and *jaz7-1* mutants.(c,d) Analysis of cross-sections taken from the base of 30 cm wild-type (c) and *jaz10-1* (d) stems. Lateral extension of the ICD is indicated by brackets. Scale bar = 200 μm; same magnification in (c) and (d).(e) Comparison of stem diameter at the base between wild-type, *jaz10-1*, *jaz10-2* and *jaz7-1* mutants.(f) Analysis of longitudinal IC extension in 25 cm stems of wild-type, *coi1-1* and *myc2-3* mutants.

We identified two lines carrying T-DNA insertions in *JAZ10*, and classified *jaz10-1* (SAIL_92_D08) as a strong allele and *jaz10-2* (GK-421G12) as a weak allele based on RT-PCR analyses (Figure S5). For *JAZ7*, a T-DNA insertion line was identified (*jaz7-1*, WiscDsLox7H11) in which the open reading frame was disrupted, suggesting that gene function is severely impaired (Figure S5). We did not observe a phenotypic alteration in the overall growth dynamics or organ shape in any of the mutants in comparison to wild-type. In contrast, analysis of IC dynamics showed that acropetal progression is enhanced in *jaz10-1*, *jaz10-2* and *jaz7-1*, with a pronounced enhancement in *jaz10-1*. Although no difference in the longitudinal IC extension was found in 2 cm stems, a significant increase in the longitudinal IC extension in 5 and 15 cm stems was observed in *jaz10-1*, and all three mutants showed a significant increase in the longitudinal IC extension in 30 cm stems (Figure 5a). No alteration was observed for other parameters in *jaz-7-1* and the hypomorphic *jaz10-2* mutant (Figure 5b,c). However, lateral ICD extension was significantly enhanced by 50% in 15 and 30 cm stems in *jaz10-1* (Figure 5b–d), and this was also the case for the total stem diameter (Figure 5e). To assess the possible role for the JA signalling pathway in cambium regulation, we analysed longitudinal extension of IC formation in *coi1-1* (Xie *et al.*, 1998) and *myc2-3* (SALK_061267, Figure S5), two mutants that are defective for JA signalling activators, and found a decrease of 25% in comparison to wild-type in both cases (Figure 5f).

**Figure 6:**
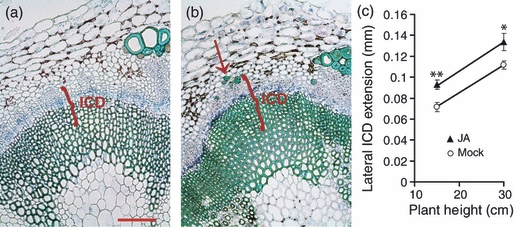
Effect of JA application on stems.(a,b) Comparison of mock- (a) and JA-treated (b) stem sections taken from the base of 30 cm plants. Lateral ICD extension is indicated by brackets. Phloem fibres are indicated by the arrow in (b). Scale bar = 100 μm; same magnification in (a) and (b).(c) Quantitative analysis of lateral ICD extension at the stem base in mock- and JA-treated plants.

Our observations show that the JA signalling repressors *JAZ10* and *JAZ7* function as repressors of secondary growth. Thus, their dynamics are comparable to those of *AUX/IAA* genes, which function as repressors of auxin signalling but are strongly induced transcriptionally by auxin itself presumably to avoid hyper-responses (Nakamura *et al.*, 2003; Wang *et al.*, 2005). Furthermore, we identified *COI1* and *MYC2*, two positive mediators of JA signalling, as promoting secondary growth. Hence, our observations are consistent with a positive role of JA signalling in cambium initiation and activity. To demonstrate that JA is directly involved in secondary growth regulation, we compared JA- and mock-treated plants with respect to the lateral ICD extension, and observed a mean 25% increase upon JA treatment (Figure 6). No effect on the dynamics of IC initiation was observed (Figure S6), which might be due to the method of JA application, which preferentially targets roots and more basal parts of plants (see Experimental Procedures). Intriguingly, we also noted JA-dependent cell wall thickening in fibre and xylem tissues, and the formation of phloem fibres, which were not present in mock-treated plants (Figure 6), suggesting that general stem stability is positively influenced by JA application. Thus, our analyses demonstrate a positive role of the JA signalling pathway in the regulation of cambium initiation and activity and in stem stability.

**Figure 7:**
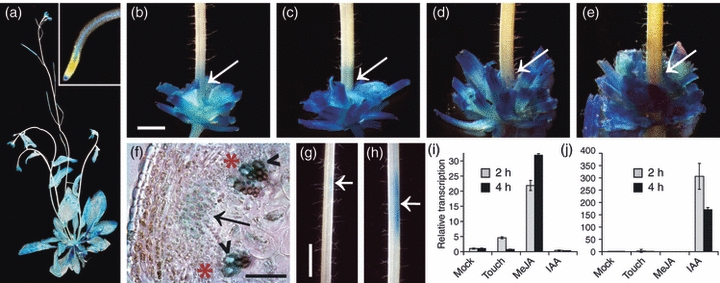
Analysis of *JAZ10* expression dynamics.(a) Whole-plant overview visualizing *JAZ10:GUS* reporter gene activity. The inset shows a close-up of a root tip.(b–e) *JAZ10:GUS* activity at the base of 2 cm (b), 5 cm (c), 15 cm (d) and 30 cm (e) inflorescence stems. Rosette leaves have been removed for clarity. Arrows indicate the stem base. Scale bar = 2 mm; same magnification in (b–e).(f) Cross-section from the base of a 2 cm plant. *JAZ10:GUS* activity is detected in xylem (arrowheads) and interfascicular regions (arrow). Primary bundles are labelled by asterisks. Scale bar = 100 μm.(g,h) Local inducibility of *JAZ10:GUS* activity by mechanical stimulation. Weak (g) and strong (h) stimulation led to different *JAZ10:GUS* activity levels (see main text for details). The site of stimulation is marked by arrows. Scale bar = 2 mm; same magnification in (g) and (h).(i,j) Quantitative RT-PCR results demonstrating the inducibility of *JAZ10* (i) and *IAA5* (j) by various stimuli. Seedlings were mock-treated, stroked by hand for 30 sec (‘touch’), sprayed with MeJA (50 μm) or sprayed with IAA (20 μm), and harvested after 2 and 4 h. *IAA5* (AT1G15570) expression was used as a positive control for IAA treatment.

To visualize JA signalling in the stem, we generated lines carrying a *JAZ10:GUS* reporter construct. Whole-plant analyses of reporter gene activity showed that the chosen promoter is active in siliques and flowers and in a variable pattern in leaves. In addition, we observed GUS activity along the stele and in the tip of the root (Figure 7a). No reporter activity was observed in stems except at the base in a region up to 2–4 mm above the rosette (Figure 7b–e). This activity was weaker in older plants, and could not be detected in the stem base of 30 cm plants (Figure 7e). An analysis of cross-sections from the stem base of 2 and 5 cm plants showed that the reporter gene activity was predominantly associated with the xylem and interfascicular regions, including the IFs. No GUS signal was detected in the cambium itself or in the cortex (Figure 7f).

To investigate to what extent *JAZ10* expression is inducible locally, stems were touched over an area of approximately 1 mm using forceps for a period of 1 min, ensuring that the tissue was not visibly damaged, and harvested after 4 h. This treatment led to induction of reporter gene activity close to the stimulated area, indicating that *JAZ10* can be induced locally by touching (Figure 7g,h). Two treatment intensities, one that did not affect the cuticle (Figure 7g) and one that damaged the cuticle (Figure 7h), led to weaker and stronger GUS activation, respectively, suggesting that the strength of mechanostimulation is reflected by *JAZ10* expression levels. Quantitative RT-PCR analyses with soil-grown seedlings confirmed that both mechanostimulation and JA treatment induce *JAZ10* expression, whereas auxin treatments had no effect (Figure 7i,j). The effect of mechanostimulation resulted in a four times higher expression of *JAZ10* after 2 h which was no longer detectable 4 h after the treatment (Figure 7i), suggesting that the response is transient. In summary, these analyses demonstrate that expression of the cambium regulator *JAZ10* is specifically inducible by mechanostimulation and JA. Furthermore, the effect of mechanostimulation on *JAZ10* expression is dose-dependent, local and transient.

**Figure 8:**
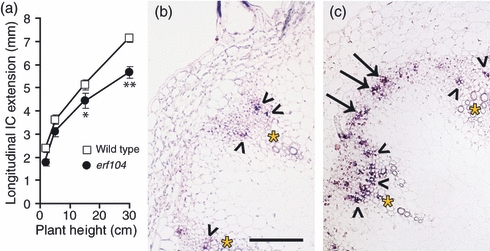
Analysis of the *erf104* mutant and H4 transcript accumulation.(a) Comparison of acropetal progression of IC initiation between wild-type and *erf104* mutants.(b,c) H4 transcript accumulation in 2 cm stems at 2.5 mm (b) and the actual stem base (c). Arrowheads indicate H4-positive cells in the FC, arrows point to interfascicular regions. Refer to Figure 3 for an overview of IC and ICD extension at this stage. Asterisks indicate primary vascular bundles. Scale bar = 100 μm; same magnification in (b) and (c).

It is well-established that mechanical forces are communicated to the cambium by ethylene signalling (Andersson-Gunneras *et al.*, 2003; Love *et al.*, 2009), and, as for JA biosynthesis, ethylene biosynthesis is stimulated by mechanical perturbations (Takahashi and Jaffe, 1984). Therefore, to determine the impact of mechanical forces on IC initiation, we analysed plants defective for the touch-inducible ethylene signalling component ERF104 (SALK_057720, Bethke *et al.*, 2009), which was identified in our transcriptional profiling (Figure 4b). Our analysis revealed a decrease in acropetal IC initiation by approximately 25% in 30 cm plants (Figure 8a), suggesting a positive role for a second stress-related hormonal pathway in IC initiation.

To determine whether putative mechanical forces at the stem base could originate from localized cell proliferations, we visualized histone H4 expression, a marker for cell division (Krizek, 1999; Barkoulas *et al.*, 2008), by RNA *in situ* hybridization. An analysis of 2 cm stems showed that cell divisions are initiated in the FC before they can be observed in interfascicular regions (Figure 8b,c), which demonstrates that the FC becomes active before the IC is initiated. This observation is in line with the possibility that intra-tissue tensions in interfascicular regions are generated by the initiation of cell proliferation in the FC.

## Discussion

We have performed a histological, molecular and genetic characterization of the dynamics of IC formation and activity at the base of the Arabidopsis shoot. IC formation is an essential process for transition from the primary to the secondary growth stage in shoot axes of dicotyledonous plants. In particular, our analysis reveals a role of JA signalling, a hormonal pathway traditionally associated with the response to wounding and mechanical perturbations, in cambium regulation.

IC initiation progresses acropetally in the elongating stem, starting from the uppermost rosette leaf, but doesn't exceed a region of about 1 cm at the stem base (Figure 2). In young stems, periclinal cell divisions are initiated in parenchyma cells between primary bundles, and are established in more and more peripheral positions during acropetal progression of this initiation and the accompanying formation of IFs. In later stages, they are also established in the starch sheath, the innermost layer of the cortex. A gradual progression of cambial activity from the FC into interfascicular regions has been reported in other species (Steeve and Sussex, 1989). We could not confirm this for the Arabidopsis stem; however, it is possible that the progression of cambial activity in Arabidopsis is too rapid to be resolved by basic histological means. Our observations are in line with the possibility that IC initiation is repressed IF cells and that the reason that IC formation occurs in parenchyma cells in some stem segments and in the cortex in others is the timing of this process with respect to IF formation (Figures 1, 2 and S2). Furthermore, the fact that the IC can be established from various cell types supports the idea of *de novo* establishment of cambium identity without the requirement for predetermination of cells present in interfascicular regions (Loomis and Torrey, 1964; Sauer *et al.*, 2006; Scarpella *et al.*, 2006).

The positive effect of mechanical forces on cambial activity and secondary growth-related gene expression has been well documented (Brown and Sax, 1962; Ko *et al.*, 2004). This and other studies (Oh *et al.*, 2003; Ko and Han, 2004; Ko *et al.*, 2004) found that a large proportion of genes preferentially expressed in secondary stems are related to stress signalling pathways and/or belong to the group of touch-inducible genes, which suggests that mechanical stimuli are involved in the regulation of gene expression in these stem segments. However, IC initiation decelerates even though the weight of the growing shoot system increases (Figures 3 and 5a), indicating that shoot weight alone is insufficient to induce IC initiation, and that instead it modulates the dynamics of secondary growth initiation and cambium activity (Ko *et al.*, 2004). In addition to shoot weight, intra-tissue forces, generated by general cell expansion or cambium activity, might contribute to tissue dynamics during secondary growth. It is tempting to speculate that, if central cells expand, peripheral tissues have to react, and cell expansion and eventually cell division are responses that may be important for avoiding tissue disruption. One example of such a process is the initiation of phellogen, a meristematic tissue that is important for cork expansion and that is established in the (sub)epidermis of the Arabidopsis hypocotyl and root (Dolan and Roberts, 1995; Chaffey *et al.*, 2002). Similarly, IC initiation could be a reaction to the FC-based tissue formation in primary vascular bundles and, potentially, produces mechanical stress in interfascicular regions. This is suggested by analysis of the timing of cell divisions at the stem base (Figure 8), which showed that cell divisions in the FC precede those in interfascicular regions. In this case, part of the initial FC-derived signal is not molecular but instead is physical in nature. Similarly, FC activity might be at least partly stimulated by internal tissue tensions generated by expansion of central parenchyma and/or the production of xylem (Brown and Sax, 1962; Hejnowicz, 1980).

The identification of *JAZ10*, *MYC2*, *COI1*, and to a minor extent *JAZ7*, as cambium regulators and the positive effect of JA application on cambium activity demonstrate that JA signalling contributes to cambium regulation (Figures 5 and 6). There is strong evidence that mechanostimulation induces JA production and the expression of JA biosynthesis genes (Chung *et al.*, 2008; Tretner *et al.*, 2008; Glauser *et al.*, 2009; Koo *et al.*, 2009). This induction is potentially triggered by cell-wall fragments, structural changes in the extracellular matrix or tensions in the plasma membrane, leading to the opening of Ca^2+^channels (Monshausen and Gilroy, 2009; Seifert and Blaukopf, 2010). Our results show that *JAZ10* in the inflorescence stem can also be induced in a local and transient manner by touching (Figure 7). In addition to its essential role as a systemic signal in plant defence and wound response (Chung *et al.*, 2008), JA signalling could play an important role in developmental processes in which tissue tensions must be released by initiating meristematic activity. Therefore, even though a negative effect of JA on cell division and in particular longitudinal root growth has been reported (Pauwels *et al.*, 2008; Zhang and Turner, 2008), a positive effect on secondary growth and stem stability seems to exist (Figures 5 and 6). Biologically, it makes sense that, upon mechanical stress, longitudinal growth is inhibited and lateral growth is promoted in order to develop a more robust plant body. This response is well known, and belongs to a wide range of mechanically induced alterations of morphology designated thigmomorphogenesis (Telewski and Jaffe, 1986; Chehab *et al.*, 2009). Currently, it is not possible to measure intra-tissue tensions, and therefore direct evidence for such tensions is missing. Molecular markers are the only tools that might serve as indicators of the presence of such tensions. Here, we found expression of the mechano-inducible *JAZ10* in interfascicular regions, which is consistent with tissue tension being present in this area. In line with this, ethylene signalling, another stress-related and mechano-inducible hormonal pathway, stimulates cambium initiation (Figure 8a) (Andersson-Gunneras *et al.*, 2003; Love *et al.*, 2009).

However, additional factors are essential for cambium initiation because JA signalling does not lead to meristem initiation *per se*. Furthermore, we did not observe the maximum level of *JAZ10* expression in the cambium, suggesting, if *JAZ10* expression reflects tissue tension, that such tensions are not strongest in the cambium itself. Auxin is one candidate for such a factor. The expression of at least one other *JAZ* gene has been shown not only to be JA- but also auxin-dependent (Grunewald *et al.*, 2009). However, we did not observe auxin inducibility for *JAZ10*, making a role of auxin upstream of *JAZ10* expression unlikely. Given the JA inducibility of auxin biosynthesis during lateral root formation (Sun *et al.*, 2009), a role for auxin signalling downstream of JA signalling is possible. We envisage a complex interplay between tissue tensions and auxin biosynthesis or transport during IC initiation. The auxin concentration may increase more quickly in cells that are located in close proximity to cells that already have an enhanced auxin content, due to auxin moving out of these cells. Thus, the proximity of interfascicular cells to the FC might be the positional trigger for initiation of IC identity (Wilson, 1978). However, it is also possible that auxin acts neither up nor downstream of JA, but in a parallel branch of the regulatory network.

Taken together, our findings indicate that the IC is initiated *de novo* in interfascicular regions by signals that presumably originate from the FC of adjacent vascular bundles, and therefore represents a secondary meristem. Moreover, we have shown a positive role for JA signalling in cambium regulation, and hypothesize that it mediates mechanical forces present in the stem, a structure that is particularly exposed to environmentally induced physical stresses. This finding provides new insights into the mechanisms underlying secondary growth, a source of a large proportion of terrestrial biomass.

## Experimental Procedures

### Plant material

*Arabidopsis thaliana* (L.) Heynh. plants of accession Columbia were used for all experiments unless stated otherwise. Plant lines not mentioned in the acknowledgements were ordered from the Nottingham Arabidopsis Stock Centre.

### Plant growth and histology

Plants were grown for 3 weeks under short-day conditions (8 h light, 16 h dark), and then shifted to long-day conditions (16 h light, 8 h dark) to induce flowering. JA treatments were performed by watering plants with either tap water (mock) or 0.5 mm jasmonic acid after moving plants to long days. Due to the asymmetric effects of side shoots on tissue patterning, only plants in which the first internode was at least 3.5 cm long were analysed. For histological analyses, stem fragments were fixed in FAA (formalin/acetic acid/alcohol) and embedded in paraffin. Subsequently, 10 μm sections were produced using a microtome, deparaffinized, stained with 0.05% toluidine blue (AppliChem, http://www.applichem.com), and fixed with Entellan (Merck, http://www.merck.com) or Dako Ultramount (Dako, http://www.dako.com) (Figure 6) on microscope slides. For quantitative analyses, at least five plants were evaluated for each data point. The standard errors of means were used to visualize variation. Data were subjected to statistical analysis, using a two-tailed independent Student’s *t* test with SPSS 15.0 software (http://www.spss.com). Significance levels of *P*<0.05, *P*<0.01 and *P*<0.001 are indicated in the figures by single, double and triple asterisks, respectively. Phloroglucinol staining and analysis of GUS reporter activity were performed as described previously (Ruzin, 1999; Scarpella *et al.*, 2004). For analysis of signal distribution in cross-sections (Figure 7), stained samples were left in 30% sucrose overnight at 4°C, then embedded in 5% low-melting-point agarose (Sigma, http://www.sigmaaldrich.com/) and sectioned using a vibratome (HM430, Microm, http://www.microm-online.com). The resulting 30 μm sections were observed using DIC optics. Alternatively, samples were embedded in Technovit 7100 (Kulzer, http://www.kulzer.com) using the manufacturer’s protocol, and 5 μm sections were produced with a microtome, fixed with Entellan and observed using dark-field optics (Figure S3).

### RNA *in situ* hybridization

RNA *in situ* hybridizations were performed as previously described (Greb *et al.*, 2003). For the H4 probe, a fragment amplified from genomic DNA using primers *H4for* (5′-TTCACATCTTTCTCACCCAAATCTACT-3′) and *H4rev* (5′- TTTCAACCGAAACTGCTGAAGC-3′) was cloned into the pGEM-T vector (Promega, http://www.promega.com/) and used as a template for transcription from the T7 promoter.

### Cloning and transgenic lines

To generate the *APL:GUS* (pTOM13) construct, a 3 kb fragment from the *APL* 5′ promoter was amplified from genomic DNA by PCR using the primers *APLfor1* (5′-ACTAGAGCTCAGCTCTTAGTTTGCTTCAACAAC-3′) and *APLrev5* (5′-ACGTCGACTGCTGCAGATCCATGGTAATCGTCTTTGGGGTCGC-3′), and a 3′ promoter fragment was amplified using the primers *APLfor5* (5′-CCATGGATCTGCAGCAGTCGACGTGATACAATTTATTAATTTTTATCTATGAGTG-3′) and *APLrev7* (5′-ACTAGGTACCGGCAAACTGTCAAATATGAAAATCG-3′). Both PCR products were cloned into pGreen0229 (Hellens *et al.*, 2000) using the *Kpn*I and *Sac*I restriction sites. Finally, the β-glucuronidase (GUS) open reading frame was cloned into *Nco*I and *Pst*I restriction sites generated between the 5′ and 3′ promoter fragments. For the *JAZ10* reporter construct, gene-specific primers were used to amplify a 2 kb fragment of the promoter region of At5g13220 on genomic Col-0 DNA (Pfu polymerase). The forward primer 5′-GCGAGCAAACCTTACGCAAA-3′ and the reverse primer 5′-ATCAAGACAGAGAGATATGGG-3′ were used with attB extensions. The fragment was then cloned into the Gateway vector pMDC162 containing a GUS gene (Curtis and Grossniklaus, 2003). Two independent transgenic lines were generated and displayed comparable reporter gene activity.

### Microarray analysis

Total RNA was isolated from stem basal (B) and internode (I) segments (Figure S3) based on a standard Trizol-based protocol, and subsequent purification was carried out using RNA-MiniElute columns (Qiagen, http://www.qiagen.com/). RNA samples were treated with RNase-free DNase (Qiagen) by column purification according to the manufacturer’s instructions. RNA quality was tested using the 260:280 nm ratio and by gel electrophoresis. For each sample, three independent RNA extractions from pools of 50 plants each were performed. cDNA production, labelling and hybridization were performed by the Arizona University Microarray Service as described at http://www.ag.arizona.edu/microarray/. Three independent hybridizations including a dye swap were performed, ensuring dye balance. Primers for performing RT-PCR validations were designed based on the oligos spotted on the array (Table S3). The cDNA template was produced utilizing a RevertAid™ H Minus First Strand cDNA Synthesis Kit (Fermentas, http://www.fermentas.com). Raw expression data have been deposited in NCBI’s Gene Expression Omnibus (Barrett *et al.*, 2009) and are accessible through GEO Series accession number GSE15446 (http://www.ncbi.nlm.nih.gov/geo/query/acc.cgi?acc=GSE15446).

### Real-time PCR quantification

RNA extraction and cDNA synthesis were performed as described above. Real-time quantitative PCR analysis was performed in a final volume of 15 μl according to the instructions in the SensiMix™ SYBR & ROX kit instruction manual (Peqlab, http://www.peqlab.com) utilizing an iQTM5 optical system (Bio-Rad, http://www.bio-rad.com/). Transformation of fluorescence intensity data into cDNA levels was performed using a standard curve constructed with a 10-fold dilution series of a single cDNA sample. The specificity of the amplification reactions was assessed using post-amplification dissociation curves. *EIF4A1* (At3g13920) was used as an internal control for quantification of gene expression based on the comparative threshold (*C*_T_) method as described by Perkin-Elmer Applied Biosystems (http://www.perkinelmer.com). For each gene, quantitative RT-PCR reactions were performed in triplicate. Primer sequences are listed in Table S3.

## SUPPORTING INFORMATION

**Figure S1**. Histological analysis of IC activity in cross-sections collected from nodal regions of cauline leaves.

**Figure S2**. Schematic representation of secondary growth dynamics in interfascicular regions at the base of the Arabidopsis shoot.

**Figure S3**. Analysis of cell identity of secondary vascular tissues, stem elongation and sampling strategy for transcriptional profiling.

**Figure S4**. Validation of the microarray data for differential gene expression.

**Figure S5**. Analysis of plant lines carrying T-DNA insertions in the *JAZ10, JAZ7* and *MYC2* genes.

**Figure S6**. Analysis of longitudinal IC extension upon JA treatment.

**Table S1**. Genes preferentially expressed in the internode.

**Table S2**. Genes preferentially expressed at the stem base.

**Table S3**. Primers used for RT-PCR analysis.

Please note: As a service to our authors and readers, this journal provides supporting information supplied by the authors. Such materials are peer-reviewed and may be re-organized for online delivery, but are not copy-edited or typeset. Technical support issues arising from supporting information (other than missing files) should be addressed to the authors.
