# Supplementary figures and images for: Analysis of secondary growth in the Arabidopsis shoot reveals a positive role of jasmonate signalling in cambium formation
Source: Plant J. 2010 Jul 14;63(5):811–22. doi: 10.1111/j.1365-313X.2010.04283.x (PMC2988407; doi:10.1111/j.1365-313X.2010.04283.x)

Figure S1

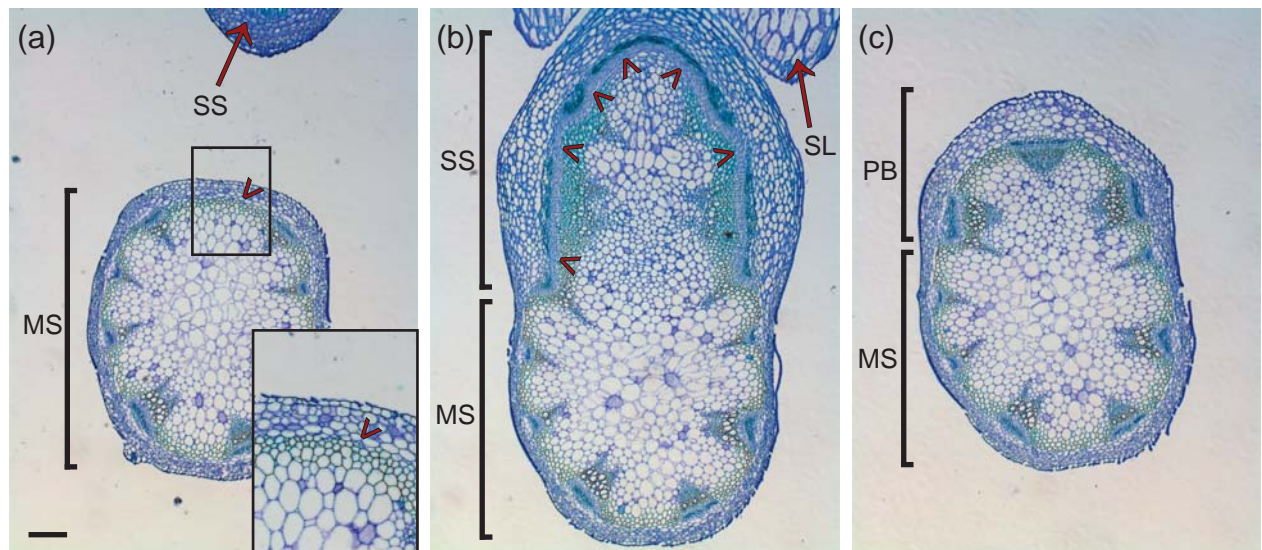

Supplement: Supplementary file 1 [file tpj0063-0811-SD1.pdf]

Figure S2

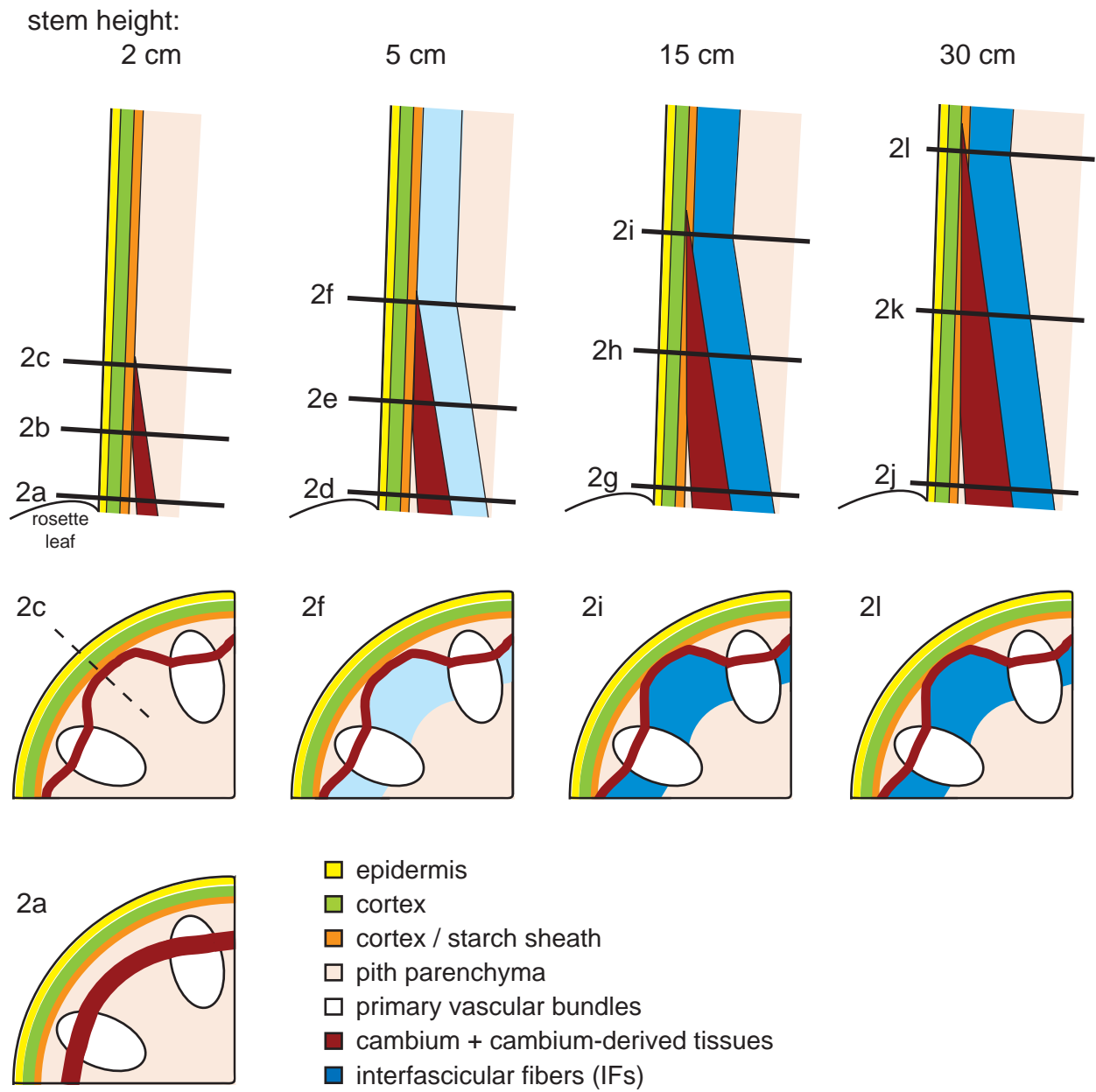

Supplement: Supplementary file 2 [file tpj0063-0811-SD2.pdf]

Figure S3

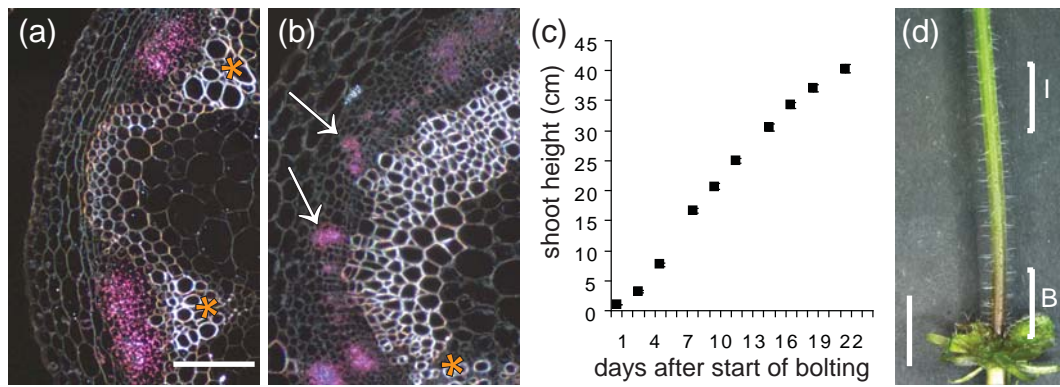

Supplement: Supplementary file 3 [file tpj0063-0811-SD3.pdf]

Figure S5

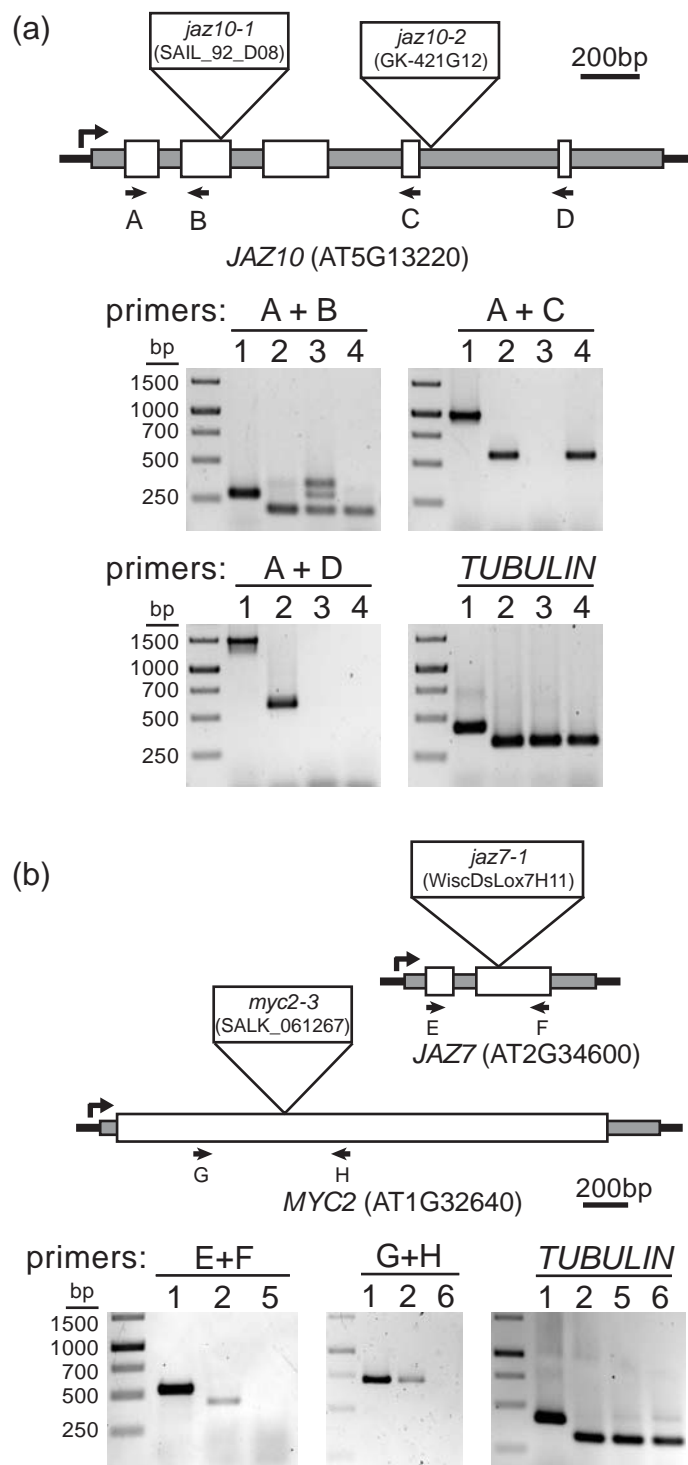

Supplement: Supplementary file 5 [file tpj0063-0811-SD5.pdf]

Figure S6

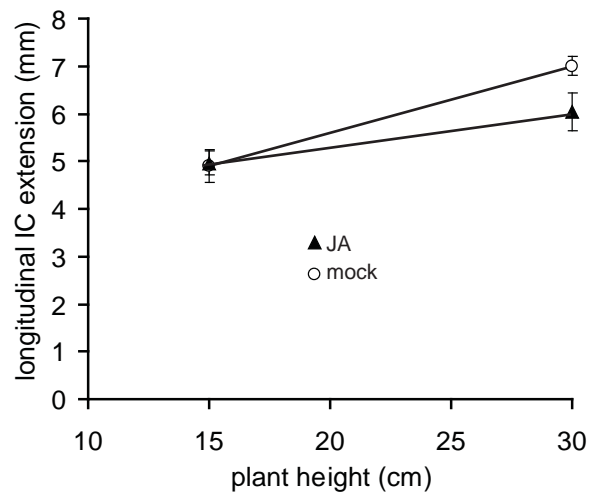

Supplement: Supplementary file 6 [file tpj0063-0811-SD6.pdf]
