# Supplementary material for: Analysis of secondary growth in the Arabidopsis shoot reveals a positive role of jasmonate signalling in cambium formation
Source: Plant J. 2010 Jul 14;63(5):811–22. doi: 10.1111/j.1365-313X.2010.04283.x (PMC2988407; doi:10.1111/j.1365-313X.2010.04283.x)
Supplement: Supplementary file 4 [file tpj0063-0811-SD4.pdf]

Figure S4

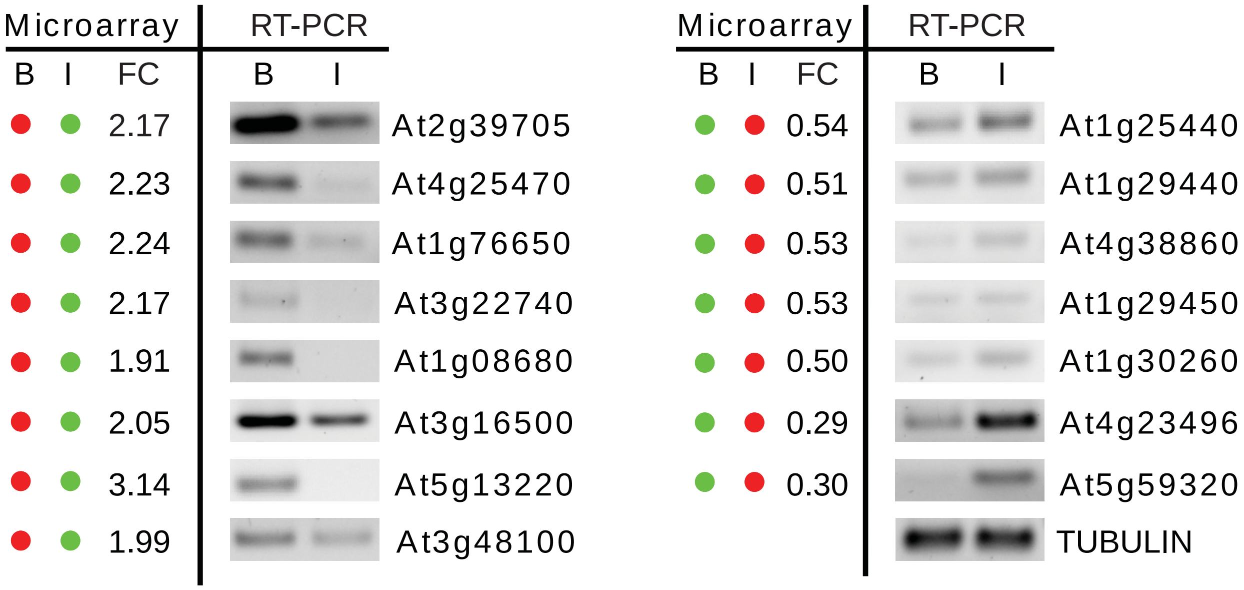
Microarray
RT-PCR
B
I
FC
B
I
2.17
At2g39705
2.23
At4g25470
2.24
At1g76650
2.17
At3g22740
1.91
At1g08680
2.05
At3g16500
3.14
At5g13220
1.99
At3g48100
Microarray
RT-PCR
B
I
FC
B
I
0.54
At1g25440
0.51
At1g29440
0.53
At4g38860
0.53
At1g29450
0.50
At1g30260
0.29
At4g23496
0.30
At5g59320
TUBULIN
