# Supplementary material for: Analysis of secondary growth in the Arabidopsis shoot reveals a positive role of jasmonate signalling in cambium formation
Source: Plant J. 2010 Jul 14;63(5):811–22. doi: 10.1111/j.1365-313X.2010.04283.x (PMC2988407; doi:10.1111/j.1365-313X.2010.04283.x)
Supplement: Supplementary file 9 [file tpj0063-0811-SD9.pdf]

Table S3

Primers for RT-PCR

Gene ID	Forward primer (5' to 3')	Reverse primer (5' to 3')
**AT2G39705**	GCCTGACTCGATCGGGAAGCGTCC	AATAAGCATGATTACGCATCGAC
**AT3G48100**	CACACATGCATGTCCTGATTC	TTGGCTAAACCAAATTGCATAAC
**AT4G25470**	TTGGCTCCGATTACGAGTCTCC	GTCAAGACCATGAGCTACCGTCG
**AT1G76650**	CGGAAACTATCACCAAAGAGG	CCATCTTCTTCTCTTCTTCGTCA
**AT1G08680**	AGCATGCAATTGCCACCCTATCC	AAGATGAATCCAAAGATGAAGCA
**AT3G22740**	AGGTCTATCGGTGGGAGAAGCAG	ACTCACGTACGACACAAAATCCT
**AT3G16500**	GAGCACAAGCTCTTCAAAGCTAGG	AGAGCTTTTAATCACACGCAGTC
**AT5G13220 (*JAZ10*)**	CTCGATTTCCTCGGACTTGAGAAG	CGATGTCGGATAGTAAGGAGATG
**AT1G25440**	AGACATGGACATCAGCGGTTGGC	TTTAACAATCAAAATGCCTTTGC
**AT1G29440**	CGCAAAGAAGCTTATGAAGATG	GCATCTAGCACTTGAGATTGACA
**AT4G38860**	CAAGGTACATCGTACCAATCTCC	CGAGATTTACATGATCCATGTAG
**AT1G29450**	CAAGTAGTGCTACTAGCTCAACC	TAGTTTCAAAGAGCATTGGGAAG
**AT1G30260**	CGAAGGTACTCGACACGATCAAGG	TTAGTTCTTGAAAACCGGAAAGG
**AT4G23496**	TCAACCTAATTGGCTCTCAATCG	AGGGATCTCCACATTACATTACG
**AT5G59320**	GCTCCATGTGCAACCTATCTATC	CGACGACGTAAGCGGCCATTTCAC
TUBULIN	TTCGTTCCTCGTGCAGTGCTCA	AAGGTAAGCATCATGCGATCTGGGT

Primers for qRT-PCR

JAZ10	ATGTCGAAAGCTACCATAGAACTCG	GCCGATGAATCGGAATTGTTTCCAGTGG
IAA5	ATCAAAGATGGCGAATGAG	CAGCTCCATCTACACTCAC
EIF4A1	ATCCAAGTTGGTGTGTTCTCC	GAGTGTCTCGAGCTTCCACTC
